# Rapid and broad detection of H5 hemagglutinin by an immunochromatographic kit using novel monoclonal antibody against highly pathogenic avian influenza virus belonging to the genetic clade 2.3.4.4

**DOI:** 10.1371/journal.pone.0182228

**Published:** 2017-08-07

**Authors:** Lam Thanh Nguyen, Kazunari Nakaishi, Keiko Motojima, Ayako Ohkawara, Erina Minato, Junki Maruyama, Takahiro Hiono, Keita Matsuno, Masatoshi Okamatsu, Takashi Kimura, Ayato Takada, Hiroshi Kida, Yoshihiro Sakoda

**Affiliations:** 1 Laboratory of Microbiology, Department of Disease Control, Graduate School of Veterinary Medicine, Hokkaido University, Sapporo, Hokkaido, Japan; 2 TAUNS Laboratories, Inc., Izunokuni, Shizuoka, Japan; 3 Laboratory of Comparative Pathology, Department of Veterinary Clinical Sciences, Graduate School of Veterinary Medicine, Hokkaido University, Sapporo, Hokkaido, Japan; 4 Research Center for Zoonosis Control, Hokkaido University, Sapporo, Hokkaido, Japan; 5 Global Station for Zoonosis Control, Global Institution for Collaborative Research and Education (GI-CoRE), Hokkaido University, Sapporo, Hokkaido, Japan; Hong Kong Institute for the Humanities and Social Sciences, HONG KONG

## Abstract

Highly pathogenic avian influenza viruses (HPAIVs) of H5 subtype have persistently caused outbreaks in domestic poultry and wild birds worldwide and sporadically infected humans. Rapid and accurate diagnosis is one of the key strategies for the control of H5 HPAIVs. However, the sensitivity of the diagnosis of H5 HPAIVs has gradually reduced due to extensive antigenic variation during their evolution. Particularly, the previously developed immunochromatographic diagnosis kit for H5 viruses, Linjudge Flu A/H5, exhibits reduced detection of H5 HPAIVs isolated in recent years. In the present study, we established a new advanced H5 rapid immunochromatographic detection kit (New Linjudge Flu A/H5) by a combination of two anti-H5 hemagglutinin monoclonal antibodies, A64/1 previously applied in the Linjudge Flu A/H5 and A32/2, a novel monoclonal antibody generated from a clade 2.3.4.4 H5 HPAIV. The new kit broadly detected all classical and recent H5 influenza viruses and showed a higher specificity and sensitivity than the original Linjudge Flu A/H5 with recently circulating H5 HPAIVs. Furthermore, the applicability of the New Linjudge Flu A/H5 was demonstrated by detecting antigens from the swabs and tissue homogenates of naturally infected birds and experimentally infected chickens with H5N6 HPAIVs belonging to the genetic clade 2.3.4.4. Our study, therefore, can provide an effective point-of-care rapid antigen detection kit for the surveillance of H5 avian influenza viruses and as a prompt countermeasure against the current widespread of the clade 2.3.4.4 H5 HPAIVs in domestic and wild birds.

## Introduction

Influenza A virus (IAV) has been classified into different subtypes according to their surface glycoproteins, hemagglutinin (HA) and neuraminidase (NA), in which 16 HA (H1–H16) and 9 NA (N1–N9) subtypes were recognized [1]. Among IAV, an H5 subtype virus has become a major global concern for the poultry industry since its first emergence in Guangdong, China in 1996, causing highly pathogenic avian influenza (HPAI) with at least 75% fatality in infected birds [2,3]. After its reemergence in 2003, the virus has consequently caused thousands of outbreaks in poultry and spread rapidly across the world via migratory wild birds [4]. In addition, the zoonotic potential of the H5 HPAIV has been recognized since the first human case of an H5 HPAIV infection in Hong Kong in 1997 [5]. To date, a total of 856 human infections with H5 HPAIVs were reported [6]. For controls of H5 HPAI in birds and H5 virus infection in humans, a simple, rapid, and accurate diagnostic tool is essential [7,8].

The typical diagnoses of IAV comprise serology or genetic identification; however, these methods are time-consuming and require appropriate facilities and biosafety [9]. In recent years, the simple and rapid immunochromatography (IC) technique, mainly based on antigen detection by monoclonal antibodies (MAbs), has been in focus because of its useful clinical diagnosis in humans and surveillance of infection in the field in birds [9]. Several IC kits for detecting IAV and their specific subtypes are widely used for these purposes [10–17]. Efficacy of IC diagnosis for IAV to detect internal nucleoproteins (NP), a highly conserved protein of IAV and large-quantity expression in cells [18], is stable. Meanwhile, detection of HA determining specific subtypes of influenza viruses remains relatively less sensitive due to lower expression in cells and large variation of the surface protein [9]. In addition, circulation of H5 HPAIVs in domestic poultry and wild birds has led to extensive antigenic diversification, so-called antigenic drift [19]. This property of the virus has caused specific and sensitive diminution of MAb reactivity against varied antigens and consequently reduced efficacy of rapid diagnosis [20,21]. A previously developed H5 IC kit manufactured by a single MAb recognizing A/duck/Pennsylvania/10218/1984 (H5N2), Linjudge Flu A/H5, reduced the sensitivity and specificity to detect recent H5 HPAIVs. Therefore the primary component of IC, MAbs specifically recognizing variable H5 HA antigens, should be formulated according to variation of viral antigenicity for more effective detection [9].

In the present study, we established an improved H5 IC rapid diagnosis kit (New Linjudge Flu A/H5) using two MAbs; A64/1, originally used in the Linjudge Flu A/H5 [10], in addition to a newly generated MAb A32/2 against a clade 2.3.4.4 H5 HPAIV. The New Linjudge Flu A/H5 showed higher specificity and sensitivity to a broad range of H5 HPAIVs isolated in recent years compared to the original Linjudge Flu A/H5 kit. In addition, its diagnostic efficacy was comparable with an influenza detection kit recognizing NP, ImunoAce Flu (NP). The diagnostic applicability of the New Linjudge Flu A/H5 was reinforced by detecting H5 HA antigens from the swabs and tissue homogenates of naturally infected birds and experimentally infected chickens with the most recent H5N6 HPAIVs in Japan. Moreover, oropharyngeal and cloalcal swabs collected from healthy chickens in commercial poultry farms were tested to demonstrate the new kit has no potential to give false-positive detection.

## Materials and methods

### Development of the IC kit to detect H5 HA antigen

Two MAbs, A64/1 and A32/2, were used in the New Linjudge Flu A/H5. The MAb, A64/1, was previously produced using a hybridoma cell line against an H5 low pathogenic avian influenza virus (LPAIV), A/duck/Pennsylvania/10218/1984 (H5N2) [22], and a novel MAb, A32/2, was prepared in a similar method against a clade 2.3.4.4 H5 HPAIV, A/chicken/Kumamoto/1-7/2014 (H5N8) [23]. The improved H5 IC kit was manufactured as described previously [10]. Briefly, the mixture of the anti-H5 HA MAbs, A64/1 and A32/2, were conjugated with colloidal gold with a proper ratio. Anti-mouse immunoglobulin antibodies and the cocktail of A64/1 and A32/2 were then immobilized onto a nitrocellulose membrane to capture antibodies in the control and test judgment regions, respectively.

### Viruses

A total of 28 strains of influenza viruses including 26 strains of IAVs, 17 strains of non-H5 viruses, 9 strains of H5 viruses, and 2 strains of influenza B viruses were used (Tables 1 and 2). These viruses were propagated in the allantoic cavity of 10-day-old embryonated chicken eggs for 30–48 h at 35°C. The infectious allantoic fluid was harvested and stored at -80°C until use.

**Table 1:** Specificity of the New Linjudge Flu A/H5, Linjudge Flu A/H5 and ImunoAce Flu (NP) with IAVs and influenza B viruses.

Viruses	Subtypes or lineages	Results ^a^		
		New Linjudge Flu A/H5	Linjudge Flu A/H5	ImunoAce Flu (NP)
A/duck/Tottori/723/1980	H1N1	-	-	6+
A/Hyogo/YS/2011	H1N1(pdm09)	-	-	6+
A/duck/Hokkaido/17/2001	H2N3	-	-	6+
A/duck/Mongolia/4/2003	H3N8	-	-	6+
A/Hokkaido/M1/2014	H3N2	-	-	6+
A/duck/Czech/1956	H4N6	-	-	6+
A/duck/Pennsylvania/10218/1984	H5N2	5+	5+	6+
A/turkey/Massachusetts/3740/1965	H6N2	-	-	6+
A/seal/Massachusetts/1/1980	H7N7	-	-	6+
A/turkey/Ontario/6118/1968	H8N4	-	-	6+
A/turkey/Wisconsin/1966	H9N2	-	-	6+
A/chicken/Germany/N/1949	H10N7	-	-	6+
A/duck/England/1/1956	H11N6	-	-	6+
A/duck/Alberta/60/1976	H12N5	-	-	6+
A/gull/Maryland/704/1977	H13N6	-	-	6+
A/mallard/Astrakhan/263/1982	H14N5	-	-	6+
A/duck/Australia/341/1983	H15N8	-	-	6+
A/black-headed gull/Sweden/5/1999	H16N3	-	-	6+
B/Hokkaido/M2/2014	B/Victoria	-	-	3+
B/Hokkaido/30-4/2014	B/Yamagata	-	-	6+

^a^ Positive/negative result of each test is indicated by +/-. Intensity of the positive test line was further recorded on a scale from + to 6+ based on visual judgments.

**Table 2:** Detection limit of the New Linjudge Flu A/H5, Linjudge Flu A/H5 and ImunoAce Flu (NP) to detect H5 HA antigens.

Viruses	Subtypes		Clades	Original virus titers^a^	Detection limits^a^		
					New Linjudge Flu A/H5	Linjudge Flu A/H5	ImunoAce Flu (NP)
A/duck/Pennsylvania/10218/1984	H5N2		-	9.6/7.5	4.8/2.8	4.8/2.8	4.6/2.6
A/chicken/Taiwan/0502/2012	H5N2		-	8.2/6.0	4.2/2.0	4.2/2.0	4.2/2.0
A/Muscovy duck/Vietnam/OIE-559/2011	H5N1		1.1	9.3/8.5	6.3/5.5	6.3/5.5	5.3/4.5
A/whooper swan/Mongolia/3/2005	H5N1		2.2	9.2/7.5	5.2/3.5	5.2/3.5	4.9/3.2
A/whooper swan/Hokkaido/4/2011	H5N1		2.3.2.1c	8.6/7.8	6.5/5.7	-^b^	3.7/2.9
A/duck/Vietnam/HU3-16/2015	H5N1		2.3.2.1c	9.8/8.0	6.1/4.3	7.8/6.0	5.8/4.0
A/chicken/Kumamoto/1-7/2014	H5N8		2.3.4.4	8.8/5.2	4.8/1.2	5.8/2.2	3.8/0.2
A/duck/Vietnam/HU1-1151/2014	H5N6		2.3.4.4	9.5/7.5	5.5/3.5	-^b^	5.8/3.8
A/black swan/Akita/1/2016	H5N6		2.3.4.4	9.1/8.0	5.4/4.3	-^b^	5.4/4.3

^a^ Original virus titers and detection limits were indicated by log_10_ EID_50_ / log_10_ TCID_50_.

^b^ “-” indicates negative result of the test with undiluted virus titer.

### Virus titration

Virus titration was performed based on the 50% tissue culture infectious dose (TCID_50_) value by using Madin–Darby canine kidney (MDCK) cells maintained in minimum essential medium supplemented with 0.3 mg/mL L-glutamine, 100 U/mL penicillin G, 0.1 mg/mL streptomycin, 8 mg/mL gentamicin and 10% calf serum. Ten-fold dilutions of viruses in serum-free minimal essential medium were inoculated onto confluent monolayers of cells and incubated at 35°C for 1 h. After 72 h of incubation at 35°C, the cytopathic effects of the cells were observed. TCID_50_ titers were calculated by the method of Read and Muench (1938). In addition to TCID_50_, the virus infectivity of H5 avian influenza viruses was measured as the 50% egg infectious dose (EID_50_) by using 10-day chicken embryos. Dilution and titer calculation were performed as described in the TCID_50_ method. The virus titration by EID_50_ and TCID_50_ was performed using the same original working aliquot of each virus.

### Evaluation of the specificity and sensitivity of the New Linjudge Flu A/H5, Linjudge Flu A/H5 and ImunoAce Flu (NP)

The detection efficacy of the present kit was compared with a human influenza commercial diagnosis kit, the ImunoAce Flu (NP antigen detection) (TAUNS Laboratories, Inc. Shizuoka, Japan), and the Linjudge Flu A/H5 kit [10]. The test procedure was performed as previously described [10]. In short, 10 μL sample solution was suspended in 90 μL of test solution (TAUNS Laboratories, Inc. Shizuoka, Japan) and the 100 μL suspension was applied to the sample port of each kit. Serial two-fold dilutions of each virus were tested. Results of the kit detection were recorded after 15 min of incubation at room temperature. A single colored line in the control judgment region (C) indicated the absence of H5 HA antigen. The concurrent presence of colored lines in both control and test judgment lines (T) indicated a positive test for H5 HA antigen in the samples. The results of antigen detection were indicated by +/-. The intensity of the positive test line was further recorded on a scale from + to 6+ (S1 Fig). To standardize the visual judgment of the test line, the optical absorbance value was measured by the fluorescent immunochromato reader DiaScan 10-T (Otsuka Electronics Co., LTD., Osaka, Japan). The detection limit showing the lowest virus titer detectable by each kit was calculated by the equivalent proportion of the original virus titers to the last dilution that was able to yield positive detection. The detection limit was expressed as log_10_ EID_50_/test and log_10_ TCID_50_/test as previously described.

In addition, oropharyngeal and cloalcal swabs collected from 25 healthy chickens in commercial poultry farms were tested to examine cross reactivity of the New Linjudge Flu A/H5 with the field specimens. These samples were also confirmed to be negative with IAV by virus isolation using embryonated chicken eggs as previously described (S1 Table).

### Swabs and tissue homogenates of naturally infected birds and experimentally infected chickens with H5N6 HPAIVs

A dead black swan and a dead whooper swan suspected of having natural infections with H5 HPAIVs were transferred to our laboratory for diagnosis. Two H5N6 HPAIVs were isolated from these birds and named A/black swan/Akita/1/2016 (H5N6) and A/whooper swan/Hokkaido/X12/2017 (H5N6), respectively [24]. Simultaneously, tissue homogenates of these birds were prepared for evaluation with the IC kits. Ten percent tissue homogenates were prepared in transport medium (minimal essential medium containing 10 000 U/mL Penicillin G, 10 mg/mL Streptomycin, 0.3 mg/mL Gentamicin, 250 U/mL Nystatin and 0.5% bovine serum albumin fraction V) as test samples and titration of infectivity. Swab samples from these naturally infected birds were not tested since multiple swabbings were formerly performed and used for emergency diagnosis and virus isolation, the subsequent swabs collected in the necropsy might not give appropriate results for kit evaluation. In addition to the natural cases, experimental infection of chickens was performed. Briefly, 12-week-old white-leghorn chickens hatched and raised in our laboratory were used in this study. Three chickens were intranasally infected with 10^8.4^ EID_50_ of A/black swan/Akita/1/2016 (H5N6). Each chicken was housed in a self-contained isolator unit at the BSL3 facility in our laboratory. All chickens were monitored every 24 hours after the inoculation according to the standard protocol [25]. After 2 days post inoculation, swabs and organs of dead chickens were collected for kit evaluation as described above. The test samples were diluted five-fold with the test solution and tested as previously described. The viral infectivity titers in the swabs and tissue homogenates were measured and expressed as log_10_ TCID_50_/test.

All experimental protocols in this study were available in the protocols.io as http://www.dx.doi.org/10.17504/protocols.io.icmcau6.

### Ethics statements

All the animal experiments were authorized by the Hokkaido University Animal Care and Use Committee (approval numbers: 13–0138) and all experiments were performed per the guidelines of the committee. All applicable international, national, and/or institutional guidelines for the care and use of animals were followed.

## Results

### Specificity of the New Linjudge Flu A/H5

The diagnostic specificity of the New Linjudge Flu A/H5 was compared with that of the original Linjudge Flu A/H5 kit and ImunoAce Flu (NP) by using a panel of 18 reference strains of IAVs including H1–H16 subtypes together with two strains of influenza B viruses (Table 1). The New Linjudge Flu A/H5 specifically detected each of the H5 influenza viruses tested and did not show cross-reactivity with any of the other HA subtypes (Table 2). The IAVs and influenza B viruses samples were positively tested with ImunoAce Flu (NP).

To demonstrate the New Linjudge Flu A/H5 had no potential to give false-positive detection, a total of 50 oropharyngeal and cloalcal swabs confirmed to be negative with IAV were used for the kit evaluation. It was evident that the New Linjudge Flu A/H5 did not yield any false positivity with the field negative samples (S1 Table).

### Sensitivity of the New Linjudge Flu A/H5

The sensitivity of the new H5 kit was then evaluated with nine strains of H5 influenza viruses including LPAIVs and HPAIVs. Serial two-fold dilutions of each virus stock were concurrently examined by the New Linjudge Flu A/H5, the Linjudge Flu A/H5 and ImunoAce Flu (NP). The detection limit shows the lowest virus titer which was detectable by the individual tests (Table 2). The result indicates that the minimal detection limit of the New Linjudge Flu A/H5 was in the range of 10^4.2^–10^6.5^ EID_50_/test against all H5 influenza viruses examined in this study. On the other hand, the Linjudge Flu A/H5 detected HA antigens from classical clade 1.1 and 2.2 H5 HPAIVs with a comparable detection limit to the New Linjudge Flu A/H5 (10^5.2^–10^6.3^ EID_50_/test). However, for recent H5 HPAIVs classified in clade 2.3.2.1c, and 2.3.4.4, the detection limit of the original Linjudge Flu A/H5 is higher and the kit could not detect several strains in these sub-clades. Furthermore, the comparable range of detection limits of the New Linjudge Flu A/H5, the Linjudge Flu A/H5 and ImunoAce Flu (NP) were observed to detect LPAIVs and HPAIVs in a clade of 2.2 viruses (Table 2). Slight difference of limit detection was observed between virus titration in EID_50_ and TCID_50_ amongst examined H5 avian influenza viruses. However, due to inefficient replication of A/chicken/Kumamoto/1-7/2014 (H5N8) strain in MDCK cells, infectivity of the virus titrated by TCID_50_ was much lower compared with EID_50_.

### Detection of H5 HA antigen from swabs and tissue homogenates of naturally infected birds and experimentally infected chickens with H5N6 HPAIVs

The applicability of the New Linjudge Flu A/H5 for the diagnosis of H5 influenza infection was demonstrated by detecting H5 HA antigen in infected animals. Two naturally infected birds, a black swan and a whooper swan, confirmed infection with H5N6 HPAIVs, and three chickens experimentally infected with an H5N6 HPAIV, A/black swan/Akita/1/2016 (H5N6) were used for kit evaluation. A dead black swan from a local zoo was found on November 15, 2016, and stored at -20°C for shipment. Necropsy was performed on November 20, 2016 in the BSL3 facility in our laboratory. Similarly, the carcass of the whooper swan, a wild bird, was found on January 15, 2017 and transferred to our laboratory for necropsy on January 17, 2017.

In addition, three chickens were intranasally challenged and died 2 days post inoculation. Swab and tissue samples were then collected from black swan and dead chickens for subsequent kit testing. Viral titration in the swabs and tissue homogenates were performed to determine diagnostic sensitivity of the kits in clinical specimens. It was clear that the New Linjudge Flu A/H5 and ImunoAce Flu (NP) could detect the presence of H5 antigen and NP antigen, respectively in most specimens of infected birds; however, the presence of H5 antigen could not be detected with the original Linjudge Flu A/H5 kit (Table 3). Interestingly, the New Linjudge Flu A/H5 could detect H5 antigen in swabs samples from infected chickens that normally contain low virus load (10^2.2^–10^3.4^ TCID_50_/test). On the other hand, all three kits evaluated in this study did not show non-specific reactivity with specimens with the absence of H5 antigen from the uninfected chicken as control (Table 3 and S1 Table). All results emphasize the applicability of the New Linjudge Flu A/H5 for on-site diagnosis.

**Table 3:** Antigen detection from the swabs and tissue homogenates of naturally infected birds and experimentally infected chickens with H5N6 HPAIVs.

Birds^a^	Infection	Swabs^b^		Tissue homogenates^b^					
		Trachea	Cloacal	Brain	Trachea	Lung	Kidney	Spleen	Colon
Black swan	Natural	NT^c^	NT^c^	3+,-,5+ (5.1)	+,-,4+ (2.1)	+,-,6+ (3.1)	+,-,5+ (3.1)	+,-,5+ (2.8)	-,-,6+ (2.1)
Whooper swan	Natural	NT^c^	NT^c^	-,-,4+ (2.1)	-,-,4+ (0.8)	+,-,5+ (2.8)	+,-,6+ (3.8)	+,-,6+ (3.3)	-,-,3+ (1.3)
Chickens	Experimental	2+,-,6+ (3.4)	+,-,6+ (3.4)	+,-,6+ (3.3)	3+,-,6+ (4.1)	4+,-,6+ (3.3)	4+,-,6+ (3.8)	5+,-,6+ (4.1)	2+,-,6+ (3.8)
	Experimental	+,-,6+ (2.2)	+,-,6+ (2.9)	+,-,4+ (1.8)	+,-,6+ (2.1)	3+,-,6+ (5.1)	+,-,6+ (3.6)	4+,-,6+ (2.8)	+,-,6+ (2.8)
	Experimental	+,-,6+ (2.9)	+,-,6+ (2.2)	+,-,6+ (3.3)	+,-,6+ (2.8)	4+,-,6+ (3.8)	2+,-,6+ (4.1)	6+,-,6+ (4.1)	+,-,6+ (3.1)
	None	-,-,- (-)	-,-,- (-)	-,-,- (-)	-,-,- (-)	-,-,- (-)	-,-,- (-)	-,-,- (-)	-,-,- (-)

^a^ The black swan and chickens were infected with A/black swan/Akita/1/2016 (H5N6). The whooper swan was infected with A/whooper swan/Hokkaido/X12/2017 (H5N6).

^b^ Result of antigen detection of the New Linjudge Flu A/H5, Linjudge Flu A/H5, ImunoAce Flu (NP). The number in parentheses is the virus infectivity as log_10_ TCID_50_/test. (-) indicates virus infectivity under limit of detection.

^c^ NT indicates samples were not tested. Since the first swabs were used for emergency diagnosis and virus isolation, the subsequent swabs collected in the necropsy might not give appropriate results for kit evaluation.

## Discussion

For the controls of H5 HPAI in birds and H5 virus infection in humans, rapid and accurate diagnosis of the causative viruses plays a vital role to facilitate subsequent countermeasures [7,8]. Multiple studies have documented that antigenicity of the H5 HPAIVs is highly divergent; several strains of H5 HPAIVs belonging to distinct clades and sub-clades impede serological diagnosis by antisera against their earlier strains [26]. The changing antigenicity of the H5 HPAIVs has reduced the sensitivity of the IC technique against specific H5 subtypes viruses. Our laboratory testing has revealed the Linjudge Flu A/H5, previously established by our laboratory, failed to detect several H5 HPAIVs isolated in recent years (Table 2). In this study, we developed a new H5 IC kit using a combination of two MAbs. The MAb, A64/1, generated from an H5 LPAIV, A/duck/Pennsylvania/10218/1984 (H5N2), which was applied in the original Linjudge Flu A/H5 and a new MAb, A32/2, generated from A/chicken/Kumamoto/1-7/2014 (H5N8), an H5 HPAIV belonging clade 2.3.4.4 [23]. The novel MAb, A32/2, was selected amongst the four generated MAbs against A/chicken/Kumamoto/1-7/2014 (H5N8) since the A32/2 exhibited broad cross-clade reactivity with recently isolated H5 HPAIVs [23]. Furthermore, an escape mutant selected by the MAb possess an amino acid substitution at the relatively conserved position in the receptor domain and the amino acid substitution was found in several H5 strains. However, this MAb could not detect a few classical strains of H5 HPAIVs and LPAIVs [23]. The combination of A32/2 and A64/1 aimed to compensate the recognizing reactivity of each MAb for broad detection of H5 influenza viruses of the new kit. The New Linjudge Flu A/H5 shows apparent improvement over the original kit in terms of sensitivity and specificity to detect more recent H5 HPAIVs (Table 2). The sensitivity of the New Linjudge Flu A/H5 was determined and compared with that of Linjudge Flu A/H5 and ImunoAce Flu (NP) by using distinct LPAIVs and HPAIVs (Table 2). The minimal detection limit of each kit was determined as both standard methods of EID_50_ and TCID_50_ [10,11,15,16]. Since, in this study, we tried to examine various strains of H5 avian influenza viruses. Each strain has different genetic backgrounds that might significantly affect virus replication in certain conditions either in chicken embryos or MDCK cells; consequently, considerable differences in EID_50_ and TCID_50_ were observed in this study (Table 2). These results suggested that virus titration assays should be appropriately selected to evaluate diagnosis efficacy of rapid detection techniques against influenza viruses. On the other hand, virus infectivity of the samples from infected birds was titrated by TCID_50_ to minimize usage of live chicken embryos. Our evaluation indicates that the detection limit of ImunoAce Flu (NP) is in the range of 10^3.7^–10^5.8^ EID_50_/test which is almost comparable to other commercial kits to detect viral NP protein [16]. Significantly, the minimal detection limit of the New Linjudge Flu A/H5, in our study, is almost equal to that of the ImunoAce Flu (NP) to detect H5 HA antigen from LPAIVs and clade 2.2 and 2.3.4.4 HPAIVs; meanwhile, NP-targeting detection kits are generally more sensitive than that of anti-HA kits when using cultured viruses [13]. The detection limit of the present kit is comparable to the commercially available kits for specific subtype influenza viruses for H5 subtype [10,12,14] or for H7 subtype [11,15]. Although, combination of two MAbs, A64/1 and A32/2, allowed significantly improved sensitivity and specificity compared to the original Linjudge Flu A/H5 to detect recently expansive H5 HPAIVs, the sensitivity level of the New Linjudge Flu A/H5 as well as the ImunoAce Flu (NP) generally remained relatively low. This poses further challenges to improve the diagnostic efficacy of our methods in terms of sensitivity enhancement.

Point-of-care applicability of the New Linjudge Flu A/H5 was examined by detecting H5 antigen from clinical specimens of infected animals. A zoo bird and a wild bird, confirmed to be naturally infected with H5N6 HPAIVs, were transferred to our laboratory for further virological diagnosis. The New Linjudge Flu A/H5 could detect H5 HA antigen from most tissue homogenates tested. Owing to considerable roles of migratory birds in the transmission of the H5 HPAIVs, the surveillance of H5 HPAI and LPAI in wild birds has become a global emphasis [27,28]. Ordinary specimens, such as oropharyngeal and cloacal swabs collected within 24 h of dead or captured birds are preferred for viral detection; other necropsied organs are generally histopathologically examined to determine the cause of infection [27]. Here, we suggested the internal organs, which normally retain high viral loads, could be used for the scrutiny of viral detection in wild birds in addition to swab samples. Early and accurate diagnosis of the New Linjudge Flu A/H5 would be significant to minimize complexity of sample handlings and shipments in case of negative cases, or to conduct proper corresponding protocols with infection of H5 HPAI in wild birds, especially in remote areas. In addition, diagnostic efficacy was evaluated by detecting H5 HA antigen in chickens intranasally infected with the isolate, A/black swan/Akita/1/2016 (H5N6). The result of visual judgment of the kit was in accordance with virus titration. In particular, the New Linjudge Flu A/H5 detected H5 HA antigen from swab samples despite low virus titer, which was elusive in previous studies [10,11,29], and highlighted the advanced improvement for the new kit. Non-specific reactivity was not observed in samples with the absence of H5 antigen, indicating the specificity of the New Linjudge Flu A/H5 in clinical diagnosis.

It was previously concluded that the point-of-care rapid antigen detection kits are appropriate for wildlife surveillance of avian influenza despite low specificity and sensitivity of the methods [30]. In this study, the New Linjudge Flu A/H5 achieves highly sensitive and specific diagnosis against H5 HPAIVs regardless of the divergence of antigenicity of newly isolated viruses. We emphasize the ability of the New Linjudge Flu A/H5 to detect H5 HA antigens from broadly circulating viruses of H5 HPAIVs belonging to clade 2.3.2.1c and 2.3.4.4 [24,28,31,32] and LPAIVs. Most recently several H5 LPAIVs, which are genetically distinct from Asia-origin lineage, evolutionarily adapted high pathogenicity and caused serial outbreaks in birds with HPAI phenotype [33]. It is conceivable to indicate that our New Linjudge Flu A/H5 could detect these occasionally emerged H5 viruses since the new kit efficiently detected their H5 LPAI prototype strains. Nevertheless, it is also necessary to further evaluate the diagnostic efficacy of the New Linjudge Flu A/H5 to detect a broader range of H5 avian influenza viruses.

Development of the New Linjudge Flu A/H5, in fact, was initiated as an adjunct tool for laboratory rapid diagnosis against H5 HPAI. The New Linjudge Flu A/H5 has been frequently used and its applicability was truly recognized by detecting recent multiple outbreaks of H5 HPAI in Japan [24]. Furthermore, the newly validated kit might carry practical significance on preparedness for a potential human pandemic caused by H5 HPAIVs. Although, diagnosis efficacy of the New Linjudge Flu A/H5 was clearly demonstrated, the result might only give preliminary conclusion on the infection and classical standard techniques such as virus isolation and antigenic and genetic characterization are essential for further diagnosis validity and virological investigation [9].

In summary, our study is the first report to evaluate diagnostic efficacy of the point-of-care rapid antigen detection method to detect H5 HPAIVs belonging to clade 2.3.4.4 which expansively spread and cause multiple outbreaks in domestic and wild birds worldwide [24,28,32]. This study revealed suitability of the New Linjudge Flu A/H5 for surveillance of H5 avian influenza viruses in domestic and wild birds and its significance for urgent control measures against H5 HPAIVs.

## Supporting information

S1 Fig Visual judgment of the New Linjudge Flu A/H5. Result of presence/absence of antigens is indicated by +/- by appearing a line in the control judgment region (C) and presence of colored lines in both control and test judgment lines (T). Intensity of the positive test line was further recorded by a scale from + to 6+.
(TIF)

S1 Table Specificity of the New Linjudge Flu A/H5 with the swab samples from 3 experimentally infected chickens and 26 healthy chickens. (DOCX)
