# Supplementary figures and images for: Rapid and broad detection of H5 hemagglutinin by an immunochromatographic kit using novel monoclonal antibody against highly pathogenic avian influenza virus belonging to the genetic clade 2.3.4.4
Source: PLoS One. 2017 Aug 7;12(8):e0182228. doi: 10.1371/journal.pone.0182228 (PMC5546692; doi:10.1371/journal.pone.0182228)

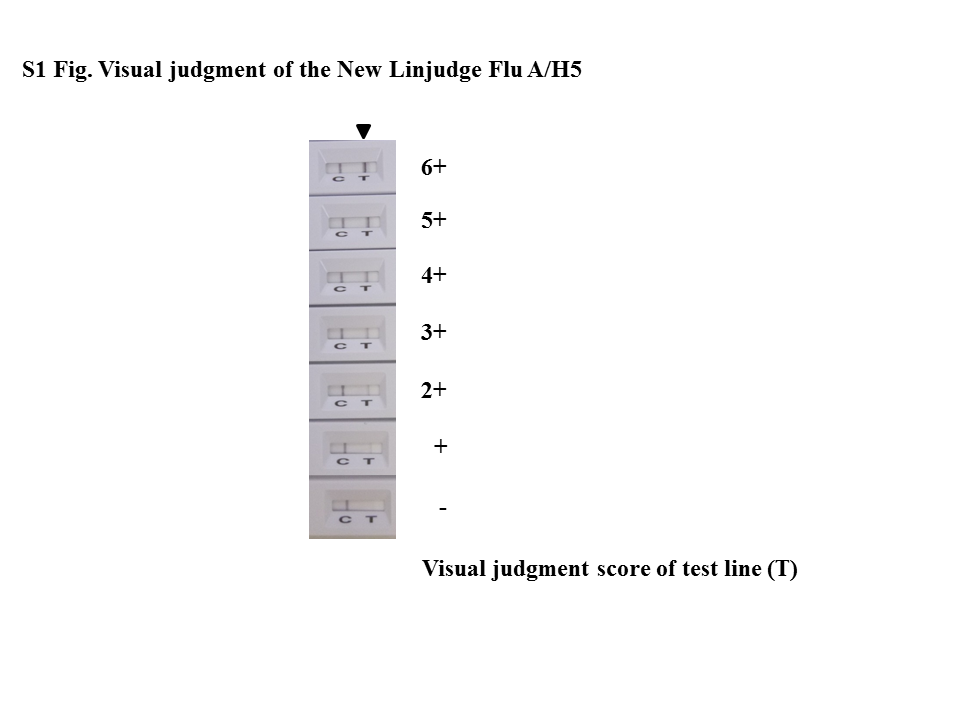

Supplement: S1 Fig — Result of presence/absence of antigens is indicated by +/- by appearing a line in the control judgment region (C) and presence of colored lines in both control and test judgment lines (T). Intensity of the positive test line was further recorded by a scale from + to 6+. (TIF) [file pone.0182228.s001.tif]
