# Supplementary material for: Rapid and broad detection of H5 hemagglutinin by an immunochromatographic kit using novel monoclonal antibody against highly pathogenic avian influenza virus belonging to the genetic clade 2.3.4.4
Source: PLoS One. 2017 Aug 7;12(8):e0182228. doi: 10.1371/journal.pone.0182228 (PMC5546692; doi:10.1371/journal.pone.0182228)
Supplement: S1 Table — (DOCX) [file pone.0182228.s002.docx]

**S1 Table. Specificity of the New Linjudge Flu A/H5 with the swab samples from 3 experimentally infected chickens and 26 healthy chickens.**

| Result of kit detection | Virus isolation  (oropharyngeal/cloalcal) | |
| --- | --- | --- |
|  | Positive^a^ | Negative^b^ |
| Positive | 3/3 | 0/0 |
| Negative | 0/0 | 26/26 |

^a^ Results of virus isolation and detection of the New Linjudge Flu A/H5 with the swab samples from experiementally infected chickens.

^b^ Result of virus isolation and detecion of the New Linjudge Flu A/H5 with the swab samples from 26 healthy chickens.
